# Interpreting mouse’s intention from analysis of prosocial behavior

**DOI:** 10.1038/s41684-025-01561-0

**Published:** 2025-05-28

**Authors:** Hiroyuki Arakawa

**Affiliations:** The Edward F. Domino Research Center, Department of Pharmacology, University of Michigan, Medical School, Ann Arbor, MI USA

**Keywords:** Zoology, Social evolution, Social behaviour

## Abstract

Recent research provides evidence of potential altruistic social behavior in mice. Laboratory mice appear to attempt to rescue an unconscious cage mate by investigating its mouth and pulling out its tongue. The finding indicates the difficulty of anthropomorphic interpretation of such impressive prosocial ability in mice.

Laboratory rodents such as mice and rats are highly social species that form complex social systems in the wild^1^. They have developed several types of social communication involving a neural system of sensory and behavioral components between conspecifics that enable individuals to recognize and identify the social and health conditions of others^2,3^. These social abilities provide promising adaptive value for group living. In a study published in *Science*^4^, the potential altruistic prosocial behavior of mice towards unconscious cage mates is reported. But do the mice intend to revive their unconscious peers? We will carefully examine the experimental procedure and data interpretation performed in this study to clarify key points for discussion on the reported ‘reviving-like’ behaviors in mice.

An unconscious (i.e., unresponsive) stimulus mouse (the same strain as the subject mice) that had been previously secured through an anesthetic injection with euthasol or inhalation with isoflurane was placed in a home cage, sprawled on its back ‘in a supine position’ as shown in Table 1, and presented to a subject mouse (C57BL/6J strain)^4^. For comparison, sleeping and dead peers were also presented to the subject mice. The ‘sleeping peer’ was anesthetized with a ketamine/xylazine compound (the procedure was not described) and placed on its belly ‘in a prone position’, a natural sleeping posture. The ‘dead peer’ was euthanized with CO_2_ and returned to the cage in ‘a supine position’, as shown in Table 1^4^, which is an uncommon posture for dead animals. Behavioral monitoring was performed when the subject mouse encountered the stimulus mouse. AI-generated ethograms for behaviors of subject mice towards stimulus mice illustrated that grooming, defined as licking the stimulus mouse, and mouth/eye interaction, defined as biting and pulling the recipient’s tongue out of its mouth and/or licking the recipient’s eye, occurred more often when confronted with an unresponsive partner than with an active (moving) partner. A limitation of these behavioral indices is that grooming was counted as the time spent in contact with the facial area of the recipients since video monitoring could not detect actual licking with the tongue. Another limitation is that mouth/eye interaction may have also included simple contact between the subject’s snout and the recipient’s mouth/snout.

**Table 1:** What factors/conditions induce reviving-like behavior in mice?

Stimulus mouse condition	Pre-treatment	Stimulus mouse position	Reviving-like behaviors (Grooming and mouth/eye interaction)
Unresponsive (anesthetized)	Euthasol or isoflurane	Supine	Expressed
Sleeping (anesthetized?)	Ketamine/xylazine (not described)	Prone	Sparse
Dead (euthanized)	CO_2_ overdose	Supine	Expressed
Active (untreated)	None	Standing/moving	Sparse

Four stimulus conditions, including unresponsive/unconscious, sleeping, dead, and active status, were compared. Reviving-like behaviors occurred when a mouse encountered either an unresponsive or dead stimulus mouse.

These behaviors are interpreted as representations of attempts to rescue unconscious peers and secure their airways. In accordance with this hypothesis, the subject mice exhibited similar responses to a ‘dead peer’ that was placed in its supine position. In contrast, they responded less to a (presumably anesthetized) ‘sleeping peer’ that was placed in the prone position (Table 1). Can mice distinguish an unresponsive (anesthetized) state from a sleeping (anesthetized) or dead (euthanized) state of a mouse peer? Although subject mice can potentially detect the remaining gas components (e.g., isoflurane or CO_2_) emanating from the mouth/body of anesthetized or euthanized stimulus mice, all these gas components are known to be aversive and typically induce avoidance reactions instead of an approach. It is thought that the overt trigger for activating these typical responses was the presence of an awkward supine position in the stimulus mice. In the wild and laboratory, mice and rats rarely exhibit a supine position during social interaction or resting^2,5^. In most cases, the dead body of a peer remains in a lying-down position or on its stomach. This unusual position may prompt the subject mice to access the mouth of the stimulus mice, stimulating the allogrooming (e.g., grooming each other) of non-resistant peers. Allogrooming has substantial adaptive functions, including thermoregulation, maintaining health by removing detritus and parasites, and controlling social and emotional conditions^6^. Furthermore, orofacial sniffing/contacts are linked to typical innate communications with familiar peers, such as whisker trimming^7^, unexplained natural prosocial behavior, and social transmission of food preference via olfactory detection of food odors and CS_2_ intestinal gas^8^. Therefore, the reported behaviors can be interpreted as a congenital drive to access the orofacial area, which facilitates allogrooming in an open mouth leading to pulling tongue and removing a stuffed foreign body.

Neural circuit studies have demonstrated that these prosocial interactions are initiated by an investigatory approach to social cues, which is regulated by the neuropeptide oxytocin (OXT) synthesized mainly in the paraventricular nucleus of the hypothalamus (PVN)^9^. In the current study, the presence of an unresponsive (anesthetized) mouse (not described if in a supine or prone position) through a wire mesh induced PVN-OXT neural activity along with an investigatory approach of the subject mouse, assessed by c-Fos neural activity marker and electrical neural recording. Although it is undoubtful that OXT has a critical role in social contexts, these data raise several concerns: (i) Why can exposure of an anesthetized mouse activate OXT neurons more strongly than that of an active partner, even if a wire mesh prevents physical contact? (ii) OXT projecting to the medial amygdala can stimulate the investigatory approach and contacts^10^; thus, the current OXT activation can induce investigatory approach, which is sufficient to stimulate subsequent mouth/eye interaction with a supine position peer. (iii) Because OXT release in extra-amygdala regions stimulates avoidance rather than approach^11,12^, it is not clear whether optogenetic stimulation of PVN-global OXT neurons can promote only approach/contact.

What lessons can we learn from experimental observations of prosocial behavior in mice? There is no doubt that mice have a complicated social system, and they exhibit prosocial behaviors, such as allogrooming, peer and parental caregiving and communal nesting^2^. By contrast, we regularly observe that territoriality-related aggressive attacks and cannibalism frequently occur during breeding and under certain stress conditions^13^. If we interpret the currently demonstrated mouse behaviors as an indication of a willingness to rescue a peer, then we must consider that cannibalism occurs driven by murderous impulsion or an attempt on life, which requires a great deal of consideration for anthropomorphic interpretation of the mouse behavior. Thus, the mere presence of the open mouths of supine peers can be enough to stimulate allogrooming and remove debris, as shown in this study. However, there is no logical evidence to suggest that it arises from the intention to improve airflow and thus revive an unconscious peer.
